# The Role of Microbiota in the Development of Cancer Tumour Cells and Lymphoma of B and T Cells

**DOI:** 10.7759/cureus.19047

**Published:** 2021-10-25

**Authors:** Garima Mamgain, Priyanka Patra, Manisha Naithani, Uttam Kumar Nath

**Affiliations:** 1 Medical Oncology and Haematology, All India Institute of Medical Sciences, Rishikesh, IND; 2 Biochemistry, All India Institute of Medical Sciences, Rishikesh, IND; 3 Biochemistry & Advanced Center of Continuous Professional Development, All India Institute of Medical Sciences, Rishikesh, IND

**Keywords:** b and t cell lymphoma, carcinogens, lymphocytes, microorganism, microbiota

## Abstract

Human body harbours enormous numbers of microbial organisms, including bacteria, viruses, and fungi which have a momentous role in well-being and illness in humans. Immune system shelters us from pathogenic bacteria, microorganisms found in human tissues have many benefits related to the functional movement of the host by regulating important procedures such as immunity, signalling, and breakdown.

Lymphocytes assume a significant part in the reaction to bacterial colonization, primarily by prompting a safe reaction to obstruction or initiation. Most immunologically occupant cells have a place with the mucosal invulnerable framework and are continually motioned by dendritic cells or other Antigen introducing cells that gather intestinal samples. Thus, Microbiome is a key contributor to developing lymphoma and specific alterations to microbiome composition could attenuate the risk.

There is an indication that microbial morphology can affect and control humanoids. The difference in the composition of these microorganisms is associated with tumour development. With the increased knowledge of the connection among the human microbiome and carcinogenesis, the use of these findings to prevent, predict or diagnose of lymphomas has attracted a great attention. In this article, we explored current knowledge of various microbial ecosystems, their connection with carcinogens and the potential for useful microorganisms to control and prevent B and T cell lymphoma.

## Introduction and background

Innumerable microorganisms live inside humans and interact with them in a reciprocal manner. When dysbiosis occurs, the environment becomes conducive to bacterial movement, fundamental resistance actuation, tissue damage, and carcinogenesis. Microbiota arrangement appears to affect both the restorative action and the symptoms of anticancer therapy. Regardless of amassing information supporting the part of microbiota piece in lymphomagenesis, neglected territories actually remain. In this review, we describe the role of the microbiota in B and T malignant lymphoma.

Overview of the human microbiota

Humanoids are home to numerous microbes that interact with the host in a mutually beneficial way, creating interactive and effective networks. “Intestinal gut microbiota” defines the downstream microbes present in gut. The bionetwork of viruses, archaea protozoa, fungi and bacteria all occupy enclosed spaces outside the body's membranes.

Bacteria contribute to host well-being through SCFA fusion, amino acids, and vitamins [1-3]. Among adult organisms, indole delays reproductive time by stimulating the genetic factors. This is the effect of lighting energy on micelle-derived indole therapy to reduce human weakness.

The microbiota is known for the nonstop active regeneration. Heterogeneous microbiota existing on dynoid surface is underdeveloped. This is due to several important factors such as age, atmospheric conditions, diet, smoking habits, antibiotic treatment, genetic characteristics, and experience of pathogenic species. There is considerable movement between changes in bacteriostatic therapy. Rise in gut microbiota with high sugar or fat therapy is an additional cause of circadian rhythm disturbance [4].

Many Western, vegan, gluten-free, omnivorous and Mediterranean regimes are considered for their capacity to control gut microbiota. Sequencing and metabolic examination techniques, for example, mass spectrometry is significant for surveying the design of microbiota and its hereditary advances [5-7].

## Review

Microbiota in health and disease

Within humanoid, trillions of microorganisms have evolved and continue to occur. Intestinal microbial imbalance is affected by a numeral of environmental settings, and it has a direct correlation to human health and disease. Important developments have confirmed that the gut microbiota is involved in essential biological processes in humans, including metabolic phenotype control, epithelial growth regulation, and innate immunity influence. Obesity, IBD, diabetes, metabolic syndrome, atherosclerosis, alcoholic liver disease (ALD), nonalcoholic fatty liver disease (NAFLD), cirrhosis, and hepatocellular carcinoma have also been connected to the human microbiota.

Probiotics, diet and microbiota

In the event we change our eating habits, the correlation between microbiome and diet indicates a change in health. Undoubtedly, According to data from the American Cancer Society's 2011 Global Cancer Facts and Figures, diets might be responsible for 30% of tumor cases in developed countries and 20% in developing countries.

Reports suggest that diet affects intestinal flora composition, and this makes effect on the immune response. Eating specific types of nourishments (fish, natural products, poultry and vegetables) may prevent occurring of cancer. Sometimes food metabolism can cause the formation of bioactive atoms. The synthesis of short-chain fatty acids acetate, butyrate and propionate is provoked by carbohydrate fermentation. In the gut epithelium, these substances can work with free fatty acid receptors to affect immune processes for example cytokines.

It has been shown by other researchers that butyrate can increase the number of close junction protein formations through AMP-activated protein kinase modulation. A portion of the portrayed activities of butyrate are enhancements of explicit favorable to apoptotic quality articulation in tumor cells and the decrease of the supportive of incendiary pathway of NF-kB. The immune response is strengthened by probiotics by increasing the cytotoxicity of natural killer cells (NK) and by activating phagocytes. The effectiveness of NK cells can also be improved when a combination of probiotics and dextran is used. In addition, the growth of IgM and IgA immunoglobulins seems to be rising with the rise in probiotics. The conformation of intestinal bacteria is altered by probiotics, for example, Oscillibacter and Prevotella, known manufacturers of anti-inflammatory substances.

Carcinogenesis and microbiota

Oncoviruses contain microscopic organisms that can straightforwardly harm DNA and modify the cell measures [8-10]. Some well-known tumor viruses introduce tumor sources into the hereditary host material. It is worth that many microbes use ingenious tricks that damage the DNA of competing bacteria. Unfortunately, host DNA that causes modifications and carcinogenesis is also suitable for this particular process. Microbial DNA can be inserted primarily through intermediate RNAs in humanoid genomes, such as the mitochondrial genome, and occurs more frequently in relatively healthy tissues than in malignant tissues [11]. Modifications produced by microbes [12,13] and microbial proteins change the DNA, it can also trigger signaling activity in the host pathway leading to cell growth.

However, some microorganisms are called tumor viruses (see Table 1). Due to ecologically diverse organisms, the underlying mediator may not be clear due to the location of the tumor, and as a result of "hit and run" behaviour after fleet behaviour cooperation, microbes may suffer cellular damage to track the host. There are rising signs that the structure of the microbial community can disturb the human immune tissue [14]. Dysfunctional microbial composition is related with reduced immunity, disease susceptibility, and the underlying disease [15,16]. Studies of sterile animals show that microbiota is a direct inducer. Toll-like receptors (TLR) [17], recognized T cells, antigen-secreting cells and lymphoid follicles [18,19] and focal field for the emergence activity of total immunomodification by increasing total antibody manufacture in addition CD4 + T-cells Resistance. . Bacteria and microbes for example lipopolysaccharides (LPS) modulate CAD, which can exacerbate the appearance of this nuclear k-factor (NF-kB), and modulate tumor-associated tenderness [20,21], invasion, progression, survival and immune responses. Microbial LPS accelerate cell production by initiating the N-terminal kinase c-Jun. Interestingly, the T17 helper cells differ from naive T cells due to fragmented filamentous microbes. Experiments have shown that these Th17 sterile mice are not found in the intrinsic layer, which is the key site of differentiation, but changes in intestinal microbiota are connected to significant differences through regulatory balance of Th17/T cells (Treg). Perhaps as epigenetic devices, these were determined by the development of factors involving transient microbiota for epigenetic transformation. Maintenance of differentiated filamentous microorganisms increased gamma interferon (IFN) production [22,23], interleukin-10, and IL-17 [24], but did not prevent sphingo monads. Bacteroidides frallis returns to Th17, which was first recognized as important for tumorigenesis, despite the involvement of the humanoid bacteria Bifidobacterium longum, Bacteroides Thytotomicron, and IFN-gamma and TNF-alpha pathways.

**Table 1:** Microbiota and chemotherapy.

Mediator	Medication	Result on tumour	References
E. coli	Gemcitabine	Reduced	42
E. coli Parabacteriodes distasonis	Doxorubicin	Reduced	42
Lactobacillus acidophilus	Cisplatin	Reduced	45,46
Germ-free mice	Platin	Reduced	44

**Figure 1:**
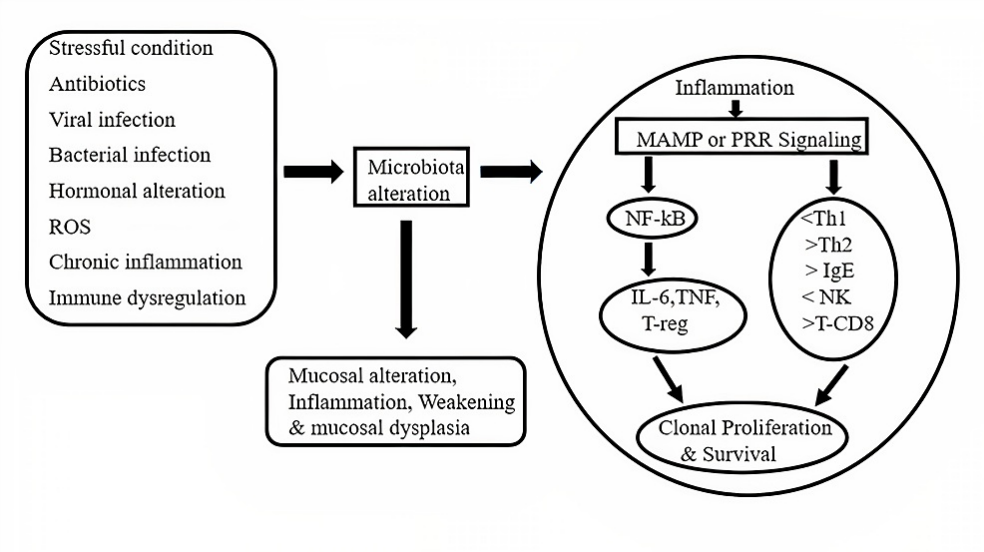
Carcinogenesis and microbiota. #Microbe-associated molecular pattern (MAMP), reactive oxygen species (ROS), pattern recognition receptor (PRR), nuclear factor-kB  (NF-kB), interleukin 6 (IL-6), tumor necrosis factor (TNF), regulatory T cells (T-reg), Type I helper T lymphocytes (Th l) , Type II helper T cells (Th 2), immunoglobulin E (IgE), natural killer (NK), CD8+ (cytotoxic) T cells (T-CD8).

As previously discussed, the intestine controls the safety of its hosts through epigenetic mutations. For example, butyrate produced by microorganisms reduces histone deacetylases 6 and 9 to mark the evolutionary number of Treg cells and further enhances acetylation at the FOXP3 gene promoter [25,26]. Metabolic and nutrient linkages almost contribute to tumor growth. Carcinogenic properties that promote tumorigenesis can include metabolic end-products [27]. Oxidative stress and interactions with “exposomes” affect the host's DNA compatibility and increase cancer risk (Figure 1) [28,29]. Many carcinogenic properties can be produced by heterologous metabolism combined with bacteriological β-glucuronidase [30]. Important examples include suboxymethane depletion and irinotecan [31,32]. Likewise, the production of harmful metabolites is caused by the formation of dietary proteins. It is associated with bacterial catabolism. All intestinal processes worsen the formation of N-nitrosorudiment, leading to DNA damage. Breakdown of aromatic amino acids leads to the development of phenol, β-cresol, indole and phenylacetic acid [33-35]. The toxicity and catabolism of important polyamines are related to oxidative stress as well as cancer (see Table 2) [36].

Microbiota in immunotherapy

Immunotherapies have radically changed the beneficial landscape of metastatic malignancies, especially immune checkpoint inhibitors (ICIs). Tumor cell IC molecules interact with their receptors on host immune cells to suppress T cell initiation, dropping the anti-tumor immune response and encouraging tumour cells to evade immunosurveillance. 

Patients' reactions to ICI action, on the other hand, differ and are often intermittent. Since a significant percentage of cancer patients are immune to ICI therapy, researchers have been working hard to find biomarkers that can consistently predict ICI therapy response. The efficiency of ICI is correlated with biomarkers, such as tumour mutational burden and tumor-infiltrating immune cells. Furthermore, immune-related adverse effects such as colitis and pneumonitis have a detrimental effect on cancer patient care, leading to ICI therapy discontinuation. Therefore, existing attempts to rise the efficiency and protection of ICI therapy and to establish suitable biomarkers are critically dependent on a thorough understanding of the ICI action process.

Some microbes create toxins that cause an inflammatory or immunosuppressive condition conducive to oncogenesis, or tumorigenic molecules. The microbial community, on the other hand, will fight cancer in the gut or at distant sites by improving the host's anti-tumor immunity. In addition, by modulating the host's local and systemic immune responses, the microbiota affects the potency and toxicity of anti-cancer treatment. The effect of the microbiota on oncogenesis and patient reaction to immunotherapeutic is addressed in this study.

Immunity

Commensal bacteria are common in the stomach. As a result, intestinal immune function prevents luminal bacteria from invading the host's tissues. Through minimising microbial-epithelial interaction (stratification) and confining invasive pathogens to the intestines and limiting their exposure to the systemic immune compartment, the immune system avoids microbial invasion. The gut microbiota, in particular, affects both local and systemic immunity. A monolayer of intestinal epithelial cells (IECs) and the underlying lamina propria make up the gut mucosa. Goblet cells develop a defensive mucus coating that is lacking in germ-free (GF) mice, covering the mucosa, indicating that microbes contribute to its creation.

Anti-microbial peptides (AMPs) are secreted by Paneth cells whose functions are stimulated by microbe immune response. Mucin and AMPs are secreted in significant numbers by IECs, which together form the mucus barrier to infection. AMPs battle a number of infections that help retain the inner mucus layer clean. Via steric hindrance and acting as releasable decoys for microbial adhesions, cell surface mucins restrict pathogen binding to IECs. Peyer's patches and a number of immune cells, such as antigen-presenting cells (APCs) [e.g., dendritic cells (DCs)], T cells, B cells, and plasma cells, are all located in the lamina propria. IgA is secreted into the intestinal lumen by plasma cells in the lamina propria.

DCs stretch transepithelial dendrites into the lumen, phagocytosing microbes specifically sample luminal antigens, and ultimately funnel antigens to hematopoietic cells. These events allow the regulated sampling of lumen antigens, triggering an adequate immune response. Via interactions with PAMPs and PRRs, the adaptive immune response to intestinal microbes is collected in the gut. DC maturation in the gut is supported by PAMPs. DCs then transfer from the gut to the mesenteric lymph nodes (mLNs), fostering the distinction between naive T cells and effector T cells (e.g., regulatory T cells [Tregs] and T helper 17 [Th17] cells).

In order to affect immune responses to remote equivalent antigens, these effector T cells travel back into the intestinal mucosa or cross the body. Tregs release anti-inflammatory cytokines (such as IL-10 and TGF-), which let the immune system to turn from pro-inflammatory to anti-inflammatory mode. Th17 cells, on the other hand, secrete pro-inflammatory cytokines (e.g., IL-17) that allow IECs to form close junctions, Paneth cells to secrete AMPs, further pro-inflammatory cytokines to be released, and the mobilisation of neutrophils from the bloodstream into the stomach. In the intestinal lamina propria, GF mice lack Th17 cells. When the small intestine is colonised by a single species of commensal microbe, they may be stimulated to generate these cells [37], meaning that microbes play an important role in Th17 cell activation. In innate and acquired immunity, TLRs play crucial roles and the interactions between TLRs and microbial elements help sustain immune homeostasis. TLRs are present on macrophages, DCs, T cells, and IECs, and they cause MYD88-dependent signalling. To generate pro-inflammatory cytokines and affect tumour initiation and growth, microbial PAMPs activate TLR signalling via the NFB and MAPK pathways.

TLR4 recognises microbe-derived lipopolysaccharide (LPS) among members of the TLR family and thus plays a critically significant role in the interactions between microbes, immunity, and oncogenesis. In mediating the impact of microbes on tumour initiation and development, tumour chemoresistance, chemotherapeutic or immunotherapeutic effectiveness and therapeutic toxicity, TLR4 signalling pathways are concerned. TLR4-targeted treatment, alone or in conjunction with microbe-targeted therapies, seems to be a promising way for treating cancer patients. 

Chemotherapy and microbial community

Chemotherapy damage usual intestinal cells and cause gastrointestinal (GI) obstruction [38]. Cytotoxic exercise in all of these treatments triggers additional immune suppression that leads to febrile neutropenia along with circulatory infections. In addition, antibiotic prevention and treatment alter the microbial community in the gastrointestinal tract [39].

Galloway-Peña et al. Intentional changes in the patient's local microflora and their clinical impact on tumor carriers using chemotherapy Indicate that many abnormalities are associated with clinical diagnosis. In addition, this information demonstrates the importance of longitudinal studies of the microbiome as a whole [40].

Unfortunately, the efficacy of chemotherapy mediators can be interfered with by direct contact with bacteria. Mass spectrometry and studies in high-performance liquid chromatography have verified biotransformation of certain chemotherapy drugs is addressed by contact with bacteria. Bacterial ability to decrease gemcitabine's antitumor effect and increase CB1 prodrug has been identified [41]. Additional information from the study model suggests complex communication between many chemotherapists and microbiota.

In vivo mechanics shows that there is a combination of platinum and cyclophosphamide (CTX) [42-44]. CTX, used to treat blood disorders, causes many microbes to harden tumors, interfere with small bowel disorders, and migrate to lymphatic tissue. This barricade breeze stimulates T-mediated anti-tumor responses and improves drug efficacy. The anti-tumor effects of platinum are dramatically summed up in sterile mice or mice with a wide range of antibiotics and condensed intestinal microflora. species are prophylactic. While it can cause antibacterial effects. Some contaminated side effects of some drugs (see Table 2) [45-47].

**Table 2:** Microbiota in cancers. Human papillomavirus (HPV), B-cell lymphoma 2 (Bcl-2), human leukocyte antigen-DR isotype (HLA-DR).

Tumor type	Microbe involved	Mechanism
Hepatocellular carcinoma	Virus of hepatitis B, Virus of hepatitis C	Stimulation of oncogene
Cholangiocarcinoma	Helminth (O. viverrini, C. sinensis)	Increased cell development Decreased cell death
Gallbladder cancer	Helicobacter spp., S. typhi	Bcl-2 up-regulation, p27 down-regulation, Augmented cell invasion
Carcinoma of esophageal squamous cells	HPV	Mucosal modifications, inflammation, weakening and mucosal dysplasia
Gastric Cancer	Klebsiella pneumoniae	Oncogenic activation
Head and neck carcinoma	Lactobacillus colehominis, Acinetobacter baumani	Mucosal alterations, inflammation, weakening and intestinal metaplasia
Lung cancer	Helicobacter pylori	Modifications with clinical-pathologic characteristics
Breast cancer	Parvimonas, HPV	Mucosal alterations, inflammation, weakening and mucosal dysplasia
Cervical cancer	Gut microbiome	Increased vaginal pH
Acute lymphoblastic leukemia	A. vaginae, Porphyromonas sp.	Oncogenic activation
Hodgkin lymphoma	HPV	Immune system dysregulation by IL-6
Non-Hodgkin lymphoma	Firmicutes, Lactobacillales, Abiotrophia, Granulicatella, etc	HLA-DR+CD4+ and HLA-DR+CD8+ T cells
Chronic lymphatic leukemia	During puberty, gut microorganisms	Immunological modifications:

The abdominal chemical toxicity of methotrexate is in part due to the production TLR4 bacterial foods for example Cif, an unknown poison of Pseudomonas aeruginosa. The appearance of TLR2 shelters the mucous membrane despite damage by methotrexate, which compensates for the appearance of ABC 1-containing protein 1, which accelerates xenophobic outflow through abdominal epithelial cells [48].

Microbiota and B and T cell lymphoma

The presence of specific microorganisms is associated with many lymphomas. Adolescent Young Adult Hodgkin's Lymphoma (AYAHL) in young adults related to exposure. AYAHL is connected with inhibition of Th1 start and improved Th2 response [49]. The deposition of intestinal bacteria in childhood is consistent with alteration Th2 to developed Th1-induced resistance pattern. In Hodgkin's lymphoma increasing interest, Th2 and IgE cytokines among AYAHL focuses on fused NK cells & cytotoxic T cells [50], resulting in difficulty with the Th2 to Th1 change. This information is based on the intestinal microflora AYAHL [51]. Cozen et al. found out if the diversity of fecal bacteria differs between AYAHL fighters. Randomized gut bacteria appear to decrease in this small study of AYAHL firefighters. Further training is required to clarify whether the decrease in microbial diversity is the cause of Hodgkin's lymphoma [52,53].

Carcinogenic diseases, for example, Epstein-Barr virus, human herpesvirus and humanoid T cell leukaemia type 1 are associated with about 12 percent of all human tumors [54]. Incidence of viral tumors, mainly lymphomas, depends on geology and depends on complex temperature and environmental conditions [55]. Oxidative stress caused by intestinal bacteria interferes with carcinogenesis and affects many pathways involved in the development of lymphoma. Mucosal-associated lymphoma of the lymph tissue (MALT) is related with the existence of Helicobacter and is designed to develop within the peripheral branch [56-58]. In the animal version infected by the relative H. felis. pylori, this accessory took first place and 154 days after infection, there was a variation in the lymph epithelium in 25 percent of mice, but there was no control group [59]. Initially, contamination with H. pylori documented and an rise in metaplasia in intestine confirmed in Gerbil [60]. An animal model reported infection with pylori [61,62].

H. pylori characterized as carcinogen (Group I carcinogenic). This is based on evidence from some papers that designed gastric cancer by funding from the International Cancer Research Agency (IAC) Staff Group in 1994 [63]. In 2009 The group evaluated a much more potent effect, determined that the group 1 chemical was persistent H. pylori contamination, and showed adequate indications that the infection was the root cause of low-quality Gastric cancer and B-motility gastric MALT lymphoma (GML). The lymphoma of MALT is highly associated with Infection with H. pylori. Looking at the 144 consecutive patients recommended by GML, it was still possible to discontinue treatment to create complete remission (CR) in the future long-term prognosis. During the interdisciplinary EI maintenance phase, 92% of participants confirmed H. pylori discontinuation. 83% After that, a complete response was obtained after seven months and 86% continued treatment after an extended period of 105 months [64].

Contact with additional types of lymphoma is still questionable. Many studies classify most diffused large B cell lymphomas (DLBCL) as H. pylori structures. In particular, DLBCL may be combined with H. pylori withdrawal. DLBCL, however, can grow speedily if not involved in H. pylori abolition, unlike MALT lymphoma. . Consequently, it is very important to identify a biomarker that can predict the gastric DLBCL site in the H. pylori structure. Kuo et al. Taiwan proposed that the appearing p-SHP2 and p-ERK signaling residues of cytotoxin-related gene A (Cag A) and Cag A in malignant B cells is related to the requirement of H. pylori. Activator targeting (BAFF) indicates MALT by activating the BCL3 and BCL10 non-H. pylori motifs of NF-rB and nuclear translocation. H. helmanii also sacrifices MALT lymphomas that migrate through endothelial-like vesicles, which may be associated with lymphocyte utilization [65]. However, these microbial lymphoma models seem to have varying outcomes and can bind to microbial and host factors [66,67]. Lymphoma may also develop from microbes, for example, Borrelia bergdorferi, Chlamydia psitacci and Campylobacter jejuni [68].

Borrelia burgdorferi infection can be caused by non-Hodgkin B-cell lymphoma, as indicated by one or two sources in Scandinavia Chlamydia psittaci, a modulator of psittacosis in animal infectious diseases, has been found in many non-Hodgkin lymphomas [69,70]. Hematopoietic illnesses, for example, persistent myeloid leukemia & persistent lymphocytic leukemia, are associated with Streptococcus bovis [71]. Interesting outcomes were obtained in creatures where the gene mutating [72] thymic lymphoma ataxia telangiectasia (Atm-/-mice) did not function [73]. Hypersensitivity changes the microbial content [74]. Barlow et al. found that in a more germ-free environment, organisms survive longer. However, when some pathogens back to their normal state, the expected tumor lifespan and duration decreased.

Prevention by targeting the microbiota

Microbiomes are now recognized as structures with more than 100 units of specific metabolic skills that break down the liver. Microbial communities can not only influence hematologic distortions through various forms of action, but also directly by metabolites, contaminants and indirectly by an intrinsic variety of adaptive resistance [75]. It remains unchanged that Could this be enough to control some types of microbes to achieve results, or could they be enhanced to reflect a combination of bacteria or modified the effectiveness of immunotherapy [76,77]. The accumulation of FMT can help achieve dangerous diseases such as acute leukemia. In a clinical medical setting, many things such as antibiotic use, malnutrition, blood flow contamination, intestinal ischemia, and defecation disorders need to be really explored, which greatly contributes to the treatment of abdominal dysbiosis and FMT [78,79]. Additional information is required to explain the cause. The basis of FMT for tumor tissues, such as modernizing intestinal microflora, improving bile acid metabolism and varying the efficacy of immunotherapy.

New approaches to changing microbiota assembly with useful lymphoma growth accessories may be used in probiotics and prebiotics. Therefore, cancer may be avoided by the whole pre- or post-probiotic microbiome, and some improvements in the microbiota may be used as an adjuvant to boost the efficacy of existing chemotherapy and immunotherapy therapies.

## Conclusions

Tumorigenesis is partially characterized, however is one of the most widely studied microbiome-related pathologies. In reality, all recent information highlights the difficulty and bidirectionality of microbiome-lymphoma relationship. As a result, the growth of lymphoma can modify the microbiome. Changes in the microbiome could influence the development of lymphoma. Microbiome interactions with B and T cell lymphoma can therefore be converted into functional applications in order to speed up diagnosis, increase effectiveness and decrease chemotherapy toxicity, and preferably avoid lymphoma by disrupting microbial carcinoma.
